# Tumor mutational burden predicts neoantigen profiles and immunotherapy response in microsatellite stable tumors across different cancer types

**DOI:** 10.3389/fimmu.2025.1582464

**Published:** 2026-01-08

**Authors:** Olesia Kondrateva, Tugce Bilgin Sonay, Inti Zlobec, Maria Anisimova

**Affiliations:** 1 Institute of Computational Life Sciences, School of Life Sciences and Facility Management, Zürich University of Applied Sciences (ZHAW), Waedenswil, Switzerland; 2 Swiss Institute of Bioinformatics (SIB), Lausanne, Switzerland; 3 Department of Molecular Life Sciences, University of Zurich, Zurich, Switzerland; 4 Institute of Tissue Medicine and Pathology, University of Bern, Bern, Switzerland

**Keywords:** immunotherapy, tumor mutational burden, TMB, microsatellite stability, MSS, neoantigens, POLE, cancer biomarkers

## Abstract

**Introduction:**

Immunotherapy has shown positive response in many patients with microsatellite instable (MSI-H) tumors, but its effectiveness in microsatellite stable (MSS) tumors remains limited. We hypothesize that tumor mutational burden (TMB) can help identify a biologically distinct subset of MSS tumors that can benefit from immunotherapy.

**Methods:**

We analyzed the molecular characteristics, including mutational landscape, mutational signatures, immune cell profiles and neoantigen load of MSS tumors with high TMB using data from colorectal cancer datasets (TCGA-COAD and TCGA-READ). After that, we extended these findings across other cancer types with MSI classification, further supporting the potential of using TMB as a biomarker for predicting immunotherapy response in MSS tumors.

**Results:**

Our results show that MSS tumors with TMB greater than 50 mutations per megabase have POLE gene mutations, which lead to hypermutation. These hypermutated tumors show immune cell signatures that are more similar to MSI-H tumors, rather than MSS tumors with low TMB. We also found that MSS tumors with high TMB have a substantially higher number of neoantigens compared to low-TMB MSS tumors, suggesting they may respond better to immunotherapy, including a high proportion of predicted high-affinity neoantigens.

**Discussion:**

These findings support the clinical relevance of TMB as a biomarker for neoantigen prediction and immunotherapy-relevant features in MSS tumors.

## 1 Introduction

Colorectal cancer (CRC) remains one of the leading causes of cancer-related deaths worldwide. This type of cancer can be categorized into two molecular subtypes: microsatellite stable (MSS) tumors and microsatellite instable (MSI-H) tumors. MSI-H is characterized by a defective DNA mismatch repair system (dMMR), which leads to an accumulation of mutations in repetitive DNA sequences, known as microsatellites. On the other hand, MSS tumors have an intact DNA mismatch repair system, which generally results in less amount of mutations.

In the last few years, significant progress has been made in understanding the molecular landscape of CRC Bilgin Sonay et al. (1)Verbiest et al. (2). This has led to more precise diagnostic and improved the choice of treatment. One of the important directions in this area involves using biomarkers to predict response to immunotherapy. Immunotherapy is treatment that uses a person’s own immune system to attack cancer cells and it has shown efficacy in different types of cancer Wang et al. (3), including colorectal cancer.

Currently, clinical studies indicate that the benefits of immunotherapy are observed mostly in patients with MSI-H tumors, whereas it appears to have limited efficacy in MSS patients Grothey et al. (4)Miyamoto et al. (5). However, MSI-H is detected in only approximately 15% of CRC tumors, making immunotherapy ineffective for the majority of CRC patients. This leads to increased interest in identifying biomarkers that can include more patients who might benefit from therapy.

One such biomarker is tumor mutational burden (TMB), which is calculated by counting the number of mutations per million base pairs of DNA. Tumors with a higher number of mutations often contain more neoantigens, which are unrecognized proteins generated by cancer cells. Neoantigens serve as targets for the immune system, potentially leading to an increased infiltration of immune cells into the tumor and, consequently, a positive response to immunotherapy Ganesh et al. (6).

MSI-H tumors typically exhibit a higher amount of mutations and a higher TMB compared to MSS tumors Timmermann et al. (7), which is one of the reasons why MSI-H tumors respond well to immunotherapy.

Although most MSS tumors display low TMB (TMB-L), there is a small, but significant subset of MSS tumors that exhibit high TMB (TMB-H). Such tumors might also be susceptible to immunotherapy, as previously suggested in the literature Fabrizio et al. (8)Marabelle et al. (9).

Therefore, the goal of this study is to investigate whether MSS tumors with TMB-H could also benefit from immunotherapy, similar to MSI-H tumors.

## 2 Results

To explore if MSS cases with TMB-H have potential to respond to immunotherapy well, we investigated datasets from The Cancer Genome Atlas Program (TCGA): TCGA-COAD (Colon Adenocarcinoma) and TCGA-READ (Rectum Adenocarcinoma).

### 2.1 POLE mutation

MSI-H samples have a high number of mutations as a result of dMMR, whereas in MSS samples, high TMB must be caused by other mutational processes. The POLE gene mutation has been previously shown to cause hypermutation in colorectal cancer samples Cancer Genome Atlas Network (10)Hino et al. (11), and we expected it to be responsible for hypermutated samples in the TCGA-COAD and TCGA-READ dataset as well.

**Figure 1:**
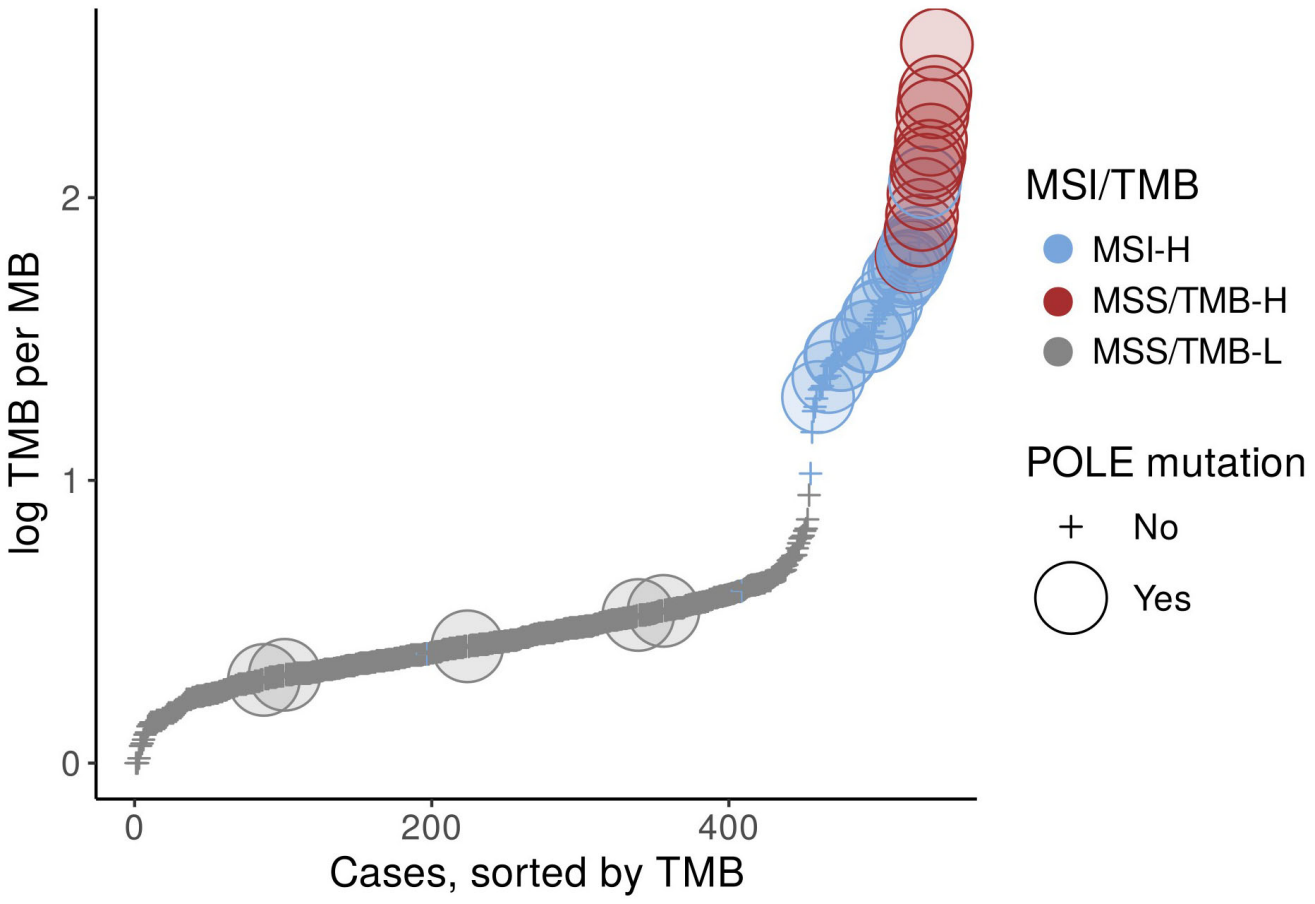
POLE gene mutations in TCGA-COAD (colon adenocarcinoma) and READ (rectum adenocarcinoma) dataset.

We found that all MSS/TMB-H samples had at least one POLE mutation, along with some MSI-H and MSS/TMB-L samples (**Figure 1**). However, just the presence or absence of POLE muation, not always cause hypermutation. To better understand whether hypermutation in MSS/TMB-H samples is driven by POLE-related mutational processes, we examined their mutational signatures.

### 2.2 Mutational signature

A mutational signature refers to a characteristic pattern of mutations in the DNA. Different factors such as exposure to certain chemicals, environmental influences, or inherent biological processes can leave distinct patterns of mutations, that could be investigated with sigminer package Tao et al. (12).

First, we calculated amount of different single base substitution (SBS) with regards of nearest nucleotides in each sample. Due to the complexity of plotting 96 parameters of SBS, we aggregated the information into five signatures for each sample. We extracted them from the SBS data using the sig extract function, which applies non-negative matrix factorization.

**Figure 2:**
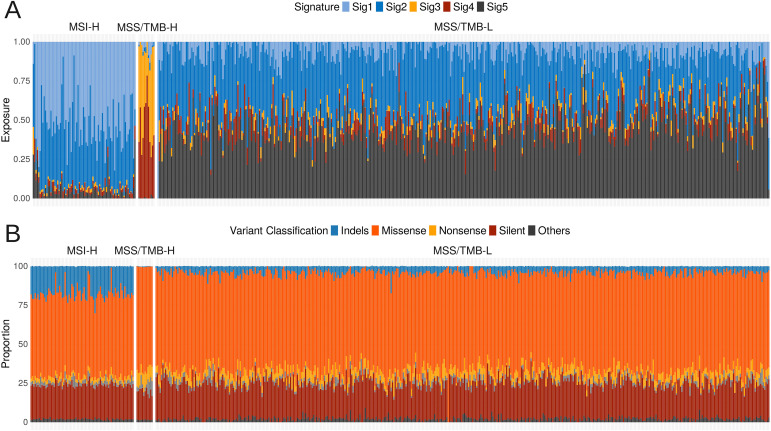
**(A)** Five extracted mutational signatures per sample across MSI-H (microsatellite instabable–high), MSS/TMB-H (microsatellite stable with high tumor mutational burden), and MSS/TMBL (microsatellite stable with low tumor mutational burden) groups. **(B)** Proportion of variant types per sample, including missense, nonsense, silent, and other mutations.

As a result, we found that each of the groups, MSI-H, MSS/TMB-H, and MSS/TMB-L, has distinct mutation signature patterns, suggesting different reasons behind mutational processes, as shown in **Figure 2A**.

**Figure 3:**
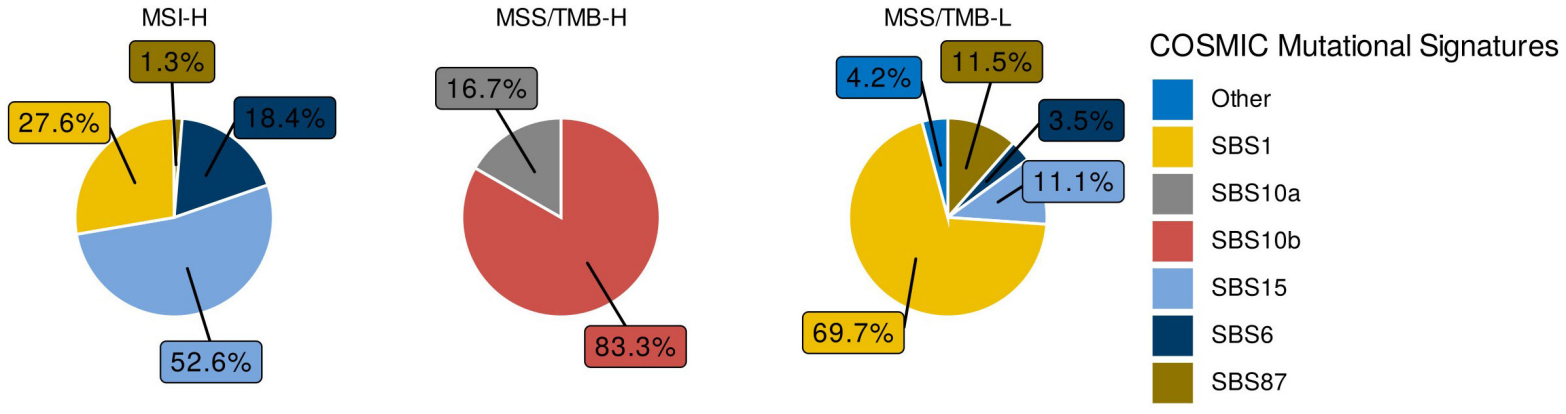
Signature with highest signal in sample for each group.

Next, we fitted the COSMIC SBS Signatures Alexandrov et al. (13) for each sample and identified the signatures with the highest exposure for each group, as shown in **Figure 3**. This allowed us to identify the mutational processes driving the mutations in each group.

In the MSS/TMB-H group, the signatures with the highest exposure were SBS10a and SBS10b, which are associated with the mutational process caused by POLE exonuclease domain mutations Li et al. (14). This finding confirms that, in the TCGA-COAD and READ datasets, POLE mutations lead to hypermutation in the MSS/TMB-H group. Our results are consistent with previous findings by Farmanbar et al. (15), who also identified SBS10 as a primary mutational signature in MSS colorectal tumors.

In addition to understanding the distinct mutational signatures calculated from SBS data, it is also important to investigate the mutational landscape of these samples. This includes identifying the types of mutations present across the groups, as different mutation types can have different impact on immunotherapy response.

### 2.3 Mutational landscape

We quantified the different mutation types and analyzed the overall mutation pattern within each group (**Figure 2B**). There was a significant difference in the amount of indel mutations between the MSI-H group and both MSS, which is a sign of deficient DNA mismatch repair Sinicrope and Sargent (16) and is consistent with characteristic of MSI-H group in colorectal cancer Maruvka et al. (17). In MSS/TMB-H samples we found that a large part of mutations is due to missense, silent and nonsense SNPs.

Overall, the mutation pattern of MSS/TMB-H is closer to MSS/TMB-L than to MSI-H. This suggests that MSS/TMB-H samples share more characteristics with other MSS samples rather than with MSI-H samples. In our analyses, the MSS/TMB-H group was distinguished by a lack of indel mutations (frameshift and inframe insertion, frameshift and inframe deletion), even when compared to the MSS/TMB-L group.

Given this distinction in the mutational landscape, to fully understand the potential response to immunotherapy in MSS/TMB-H group, we need to investigate the tumor microenvironment. In particular, we need to analyze immune cell infiltration, as the presence of immune cells within the tumor can significantly influence how the tumor responds to immunotherapy.

### 2.4 Immune cell infiltration

The TIL% stands for tumor-infiltrating lymphocyte percentage, which measures the proportion of lymphocytes that have infiltrated into a tumor. High levels of immune cell infiltration, especially a high TIL%, can indicate an active immune response Hodi and Dranoff (18) and suggest a better response to immunotherapy Ganesh et al. (6). Therefore, we investigated these factors for each group.

**Figure 4:**
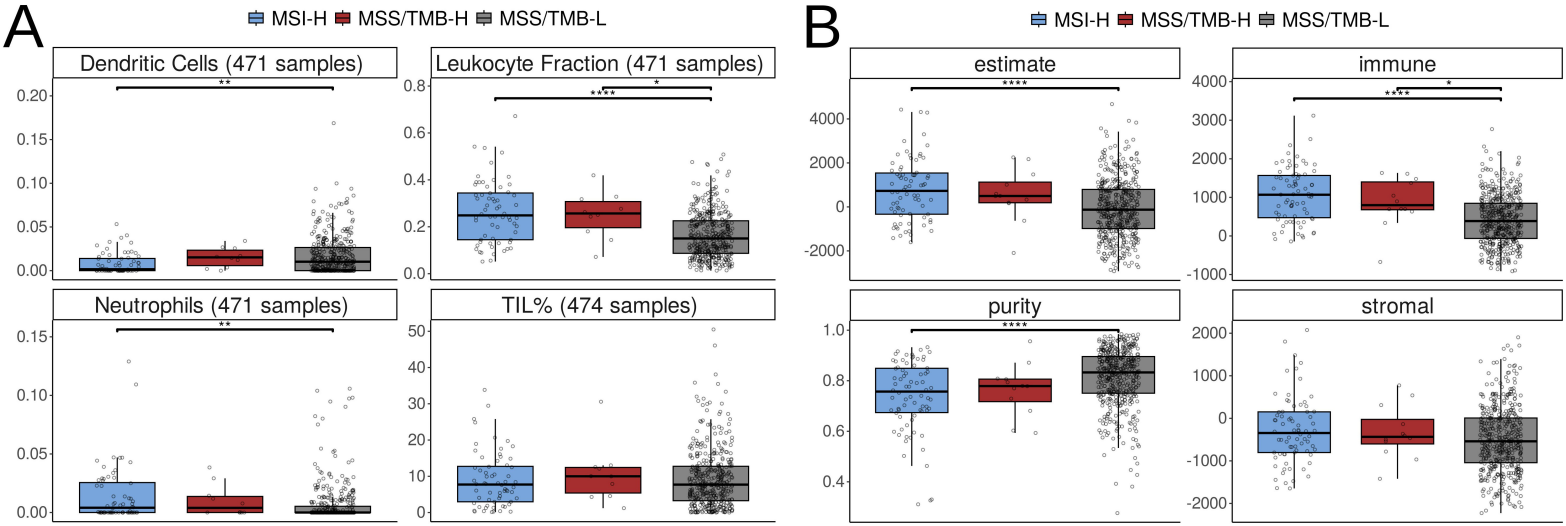
Assessing lymphocyte infiltration in colorectal cancer genomes across MSI-H (microsatellite instabable–high), MSS/TMB-H (microsatellite stable with high tumor mutational burden), and MSS/TMBL (microsatellite stable with low tumor mutational burden) groups. **(A)** The assessment of different cell types in the image data of tumor of TCGA-COAD (colon adenocarcinoma) and READ (rectum adenocarcinoma). The numbers of available samples for each cell type are indicated in the titles of the individual plots. **(B)** A comparison of ESTIMATE Scores, where stromal is stromal signature, immune is an immune signature, estimate is a score calculated by combining the stromal and immune scores, and purity indicates the tumor purity.

We used two approaches to estimate immune cell infiltration: first, by analyzing image data from tissue slides from study by Saltz et al. Saltz et al. (19), and second, by analyzing bulk gene expression using the ESTIMATE algorithm Yoshihara et al. (20). The results are shown in **Figure 4**.

There were no significant differences in TIL% calculated from image data between any of the groups, despite expectations that MSI-H tumors should show higher lymphocyte levels. This might indicate that the tissue slides used in the analysis did not capture enough information, potentially missing regions with immune cell infiltration.

There were significant differences between the MSS/TMB-H and MSS/TMB-L groups in immune signatures based on gene expression data. This difference may be primarily driven by leukocytes, as we also observed a significant difference in leukocyte levels. The MSI-H group, in addition to leukocytes, showed significant differences in dendritic cells and neutrophils.

Overall, the MSS/TMB-H group displayed an immune profile more similar to MSI-H than to MSS/TMB L. However, the signal remains weak, as not all immune parameters showed clear differences across the groups.

### 2.5 Neoantigen load

The neoantigen load is a potentially important biomarker for predicting responses to immunotherapy across various cancer types Zou et al. (21) Xie et al. (22), including colorectal cancer.

**Figure 5:**
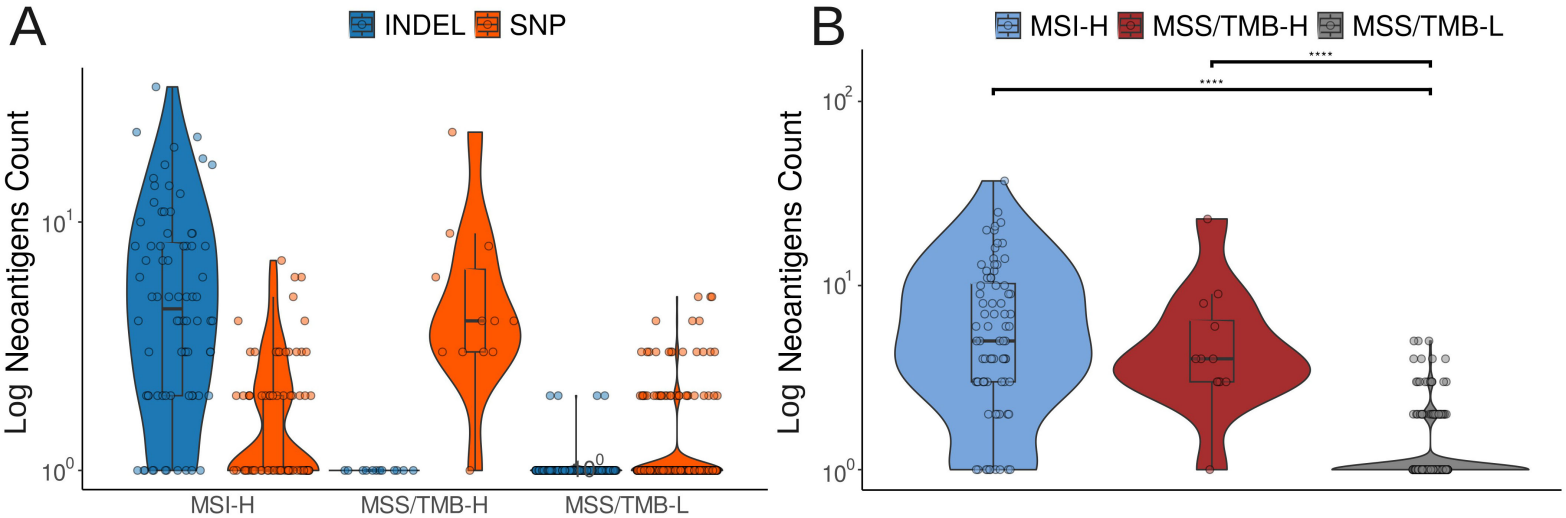
**(A)** Number of shared neoantigens that arise from indels (insertions or deletions) or SNPs (single nucleotide polymorphisms). **(B)** Total load of common neoantigen observed in each group.

First, we investigated common neoantigens, which are neoantigens that reoccur across different samples of colorectal cancer. We used a list of common neoantigens from an existing study Chen et al. (23) to compare the groups. The results showed that MSS/TMB-H samples exhibited patterns similar to MSI-H samples (**Figure 5**). The key distinction was that most neoantigens in MSI-H samples were derived from indel mutations, whereas in MSS/TMB-H, they mostly originated from missense single nucleotide polymorphisms (SNPs).

**Figure 6:**
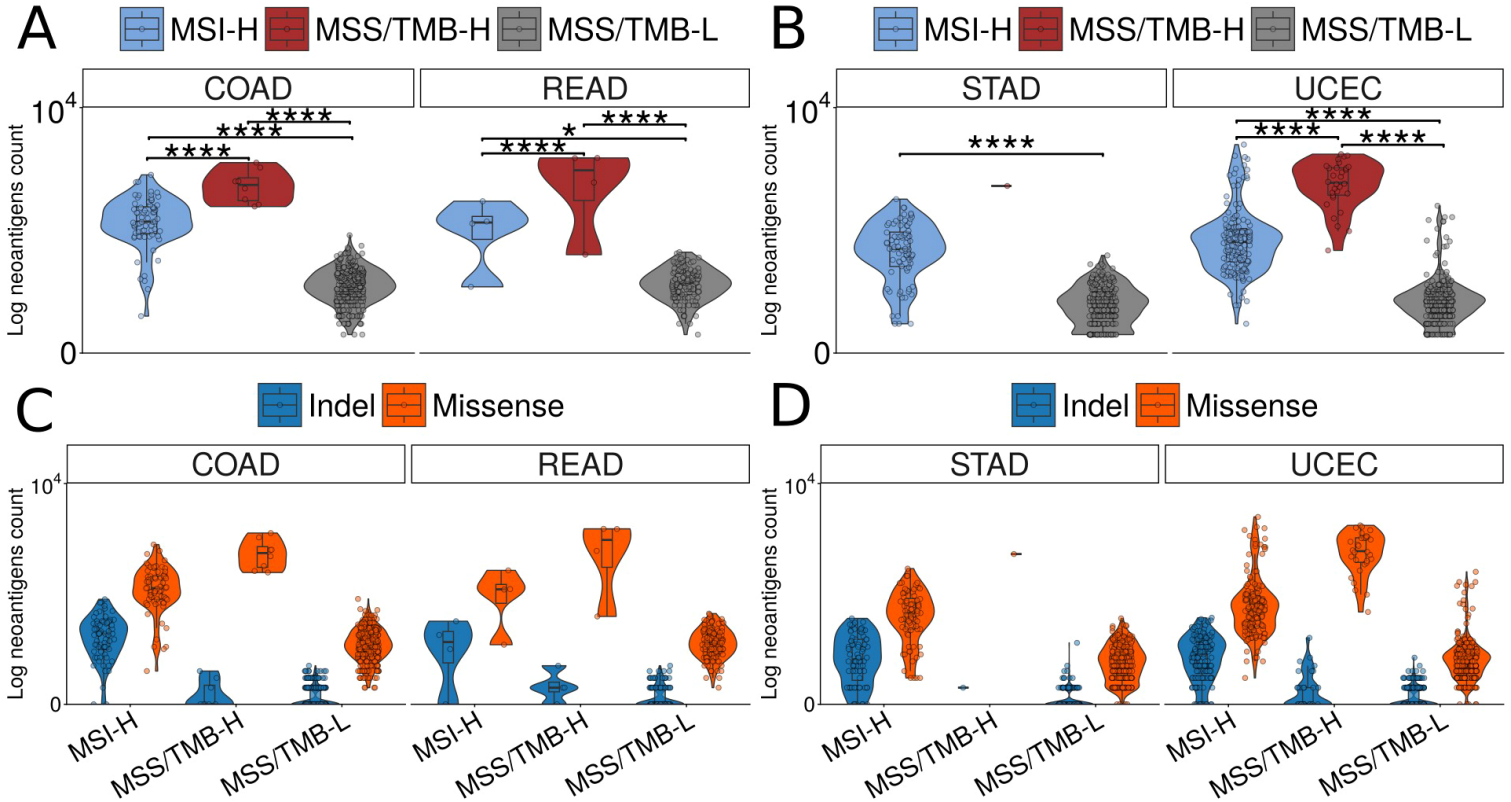
Comparison of predicted neoantigen counts across molecular subtypes and mutation types in TCGA datasets. Log-scaled total neoantigen counts per sample across MSI-H (microsatellite instabable–high), MSS/TMB-H (microsatellite stable with high tumor mutational burden), and MSS/TMBL (microsatellite stable with low tumor mutational burden) groups. **(A)** Results for TCGA-COAD (colon adenocarcinoma) and TCGA-READ (rectum adenocarcinoma) datasets; **(B)** Results for TCGA-UCEC (uterine corpus endometrial carcinoma) and TCGA-STAD (stomach adenocarcinoma) datasets. Number of peptides leading to neoantigens, grouped by mutation type, that include indel (insertion and deletions) and missense types. **(C)** Counts for TCGA-COAD and TCGA-READ; **(D)** Counts for TCGA-UCEC and TCGA-STAD.

To validate these findings, we calculated potential neoantigens for each mutation in individual samples using the pVAC-seq pipeline Hundal et al. (24). As a result, we found that the MSS/TMB-H group had an even higher number of predicted neoantigens, with most of them still coming from missense mutations, consistent with the previous findings (**Figure 6**).

We analyzed the TCGA-COAD and TCGA-READ datasets separately, as we observed differences between the datasets, with TCGA-READ samples generally showing a lower neoantigen load across all groups compared to TCGA-COAD.

We also looked for shared neoantigens. However, in the COAD dataset, we found only one shared neoantigen between MSS/TMB-H and MSI-H. This limited overlap may be due to different mutational processes in MSS/TMB-H versus MSI-H and the small number of MSS/TMB-H samples. (**Supplementary Figure S17**).

**Figure 7:**
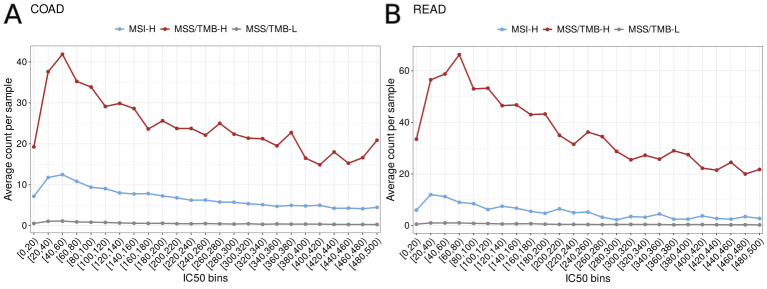
Distribution of predicted neoantigens per sample for half maximal inhibitory concentration bins (IC50) for MSI-H (microsatellite instabable–high), MSS/TMB-H (microsatellite stable with high tumor mutational burden), and MSS/TMB-L (microsatellite stable with low tumor mutational burden) groups in **(A)** TCGA-COAD (colon adenocarcinoma) and **(B)** TCGA-READ (rectum adenocarcinoma) datasets. The y-axis shows the average number of neoantigens per sample in each IC50 bin.

Although the MSS/TMB-H group has more neoantigens overall than the MSS/TMB-L and MSI-H groups, this does not always mean they are more likely to trigger an immune response. To better understand the quality of the neoantigens, we looked at how they are spread across different binding strength categories, based on IC50 values. We observed that MSS/TMB-H group had a higher number of strong binders then other groups (**Figure 7**).

**Figure 8:**
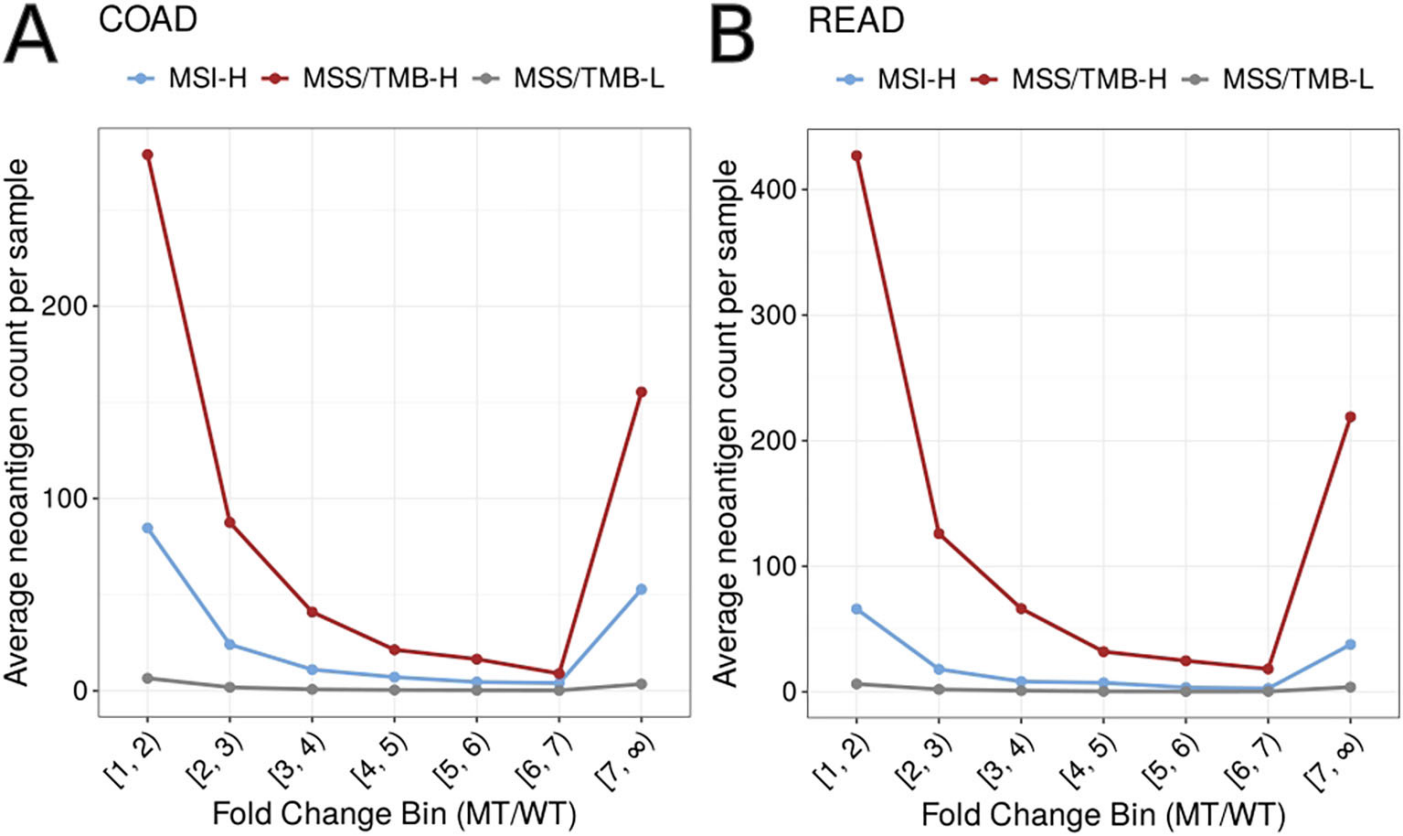
Distribution of fold change between mutant (MT) binding score and wild-type (WT) in predicted neoantigens across molecular subtypes in **(A)** TCGA-COAD (colon adenocarcinoma) and **(B)** TCGA-READ (rectum adenocarcinoma). The y-axis shows the average number of neoantigens per sample in each fold change bin.

Additionally, we evaluated fold changes of IC50 between wild-type and mutation-type across subtypes (**Figure 8**). These results suggest that MSS/TMB-H tumors often produce neoantigens with stronger predicted binding to MHC-I compared to the wild-type versions. This means that many of their neoantigens are likely to be newly presented and not recognized as self, which may increase their chance of being noticed by the immune system.

We also assessed Spearman correlations between neoantigen counts (binned by IC50) and immune cell infiltration in the COAD and READ dataset (**Supplementary Figure S16**). No strong correlations were observed, except a moderate association with leukocyte fraction in MSI-H and MSS/TMB-L groups. This suggests that neither neoantigen quantity nor binding strength alone explains variation in immune infiltration.

Finally, we examined which genes contributed most strongly to the predicted neoantigens. We observed that mutations in KRAS, TP53, and APC contributed the most in MSS/TMB-L, consistent with their roles as key driver genes for colorectal cancer. In MSI-H tumors, the highest number of candidate neoantigens was generated by mutations in the LRP1, MDN1 VPS13B, TRRAP genes. For the MSS/TMB-H group POLE, BIRC6, DST, CNOT1 contributed the most (**Figure 9**).

### 2.6 Survival analysis

We wanted to see if the differences in neoantigen load between molecular subtypes also affect patient survival. For this, we looked at overall survival in MSI-H, MSS/TMB-H, and MSS/TMB-L groups using TCGA data but did not found significant results. Patients with MSI-H tumors showed a trend toward better survival compared to those with MSS/TMB-L tumors in the TCGA-COADREAD dataset, but it was not significant. The MSS/TMB-H group did not show a clear trend in survival. This is likely because this group had fewer samples and limited follow-up information. The Kaplan–Meier plots for this analysis are shown in **Supplementary Figure S15A**.

### 2.7 Validation and extension to other cancers

We decided to validate our findings and assess the potential for extending these observations to other cancer types. We performed a similar analysis on the TCGA-UCEC (Uterine Corpus Endometrial Carcinoma) and TCGA-STAD (Stomach Adenocarcinoma) datasets, which also have MSI classifications. First of all, we identified MSS/TMB-H samples and then we examined their mutational profiles, POLE mutation status, mutational signatures, immune cell infiltration, and neoantigen load **Figures 6C, D**; (**Supplementary Figures S8**–**S14**).

In the TCGA-STAD dataset, we found only one MSS/TMB-H sample. However, the TCGA-UCEC dataset contained a substantially larger number of MSS/TMB-H samples compared to the TCGA-COAD and TCGA-READ datasets. Interestingly, all MSS/TMB-H samples in UCEC contained POLE mutations. However, an analysis of mutational signatures showed that not all these samples have a mutational process typically associated with POLE mutations. Beside that, we found that some MSS/TMB-L samples also carried POLE mutations and displayed the main mutational signature associated with POLE.

Despite these differences, the groups in the UCEC and STAD datasets showed patterns similar to those observed in the COAD and READ datasets. Specifically, MSS/TMB-H samples were characterized by a large number of missense and nonsense mutations with an absence of indels, consistent with our earlier findings. While there was no significant difference in TIL% score between the MSS/TMB-H and MSS/TMB-L groups, immune signature from the ESTIMATE algorithm and the leukocyte fraction level from image data were higher in MSS/TMB-H samples compared to MSS/TMB-L samples.

However, in the TCGA-UCEC cohort, MSS/TMB-H samples appeared to have better survival outcomes than MSI-H tumors, though this was not statistically significant (**Supplementary Figure S15B**).

These findings from the TCGA-UCEC and TCGA-STAD datasets support the patterns observed in the TCGA-COAD and TCGA-READ datasets. It validates the association between high tumor mutational burden and immune activity, and supports the potential responsiveness of these tumors to immunotherapy.

## 3 Materials and methods

### 3.1 Data

The data analyzed in this study were obtained from the TCGA projects, including gene expression, mutation information, and tumor images. A summary of the number of samples in each MSI group can be found in **Table 1**. For all datasets, we removed FFPE (formalin-fixed paraffin-embedded) samples. All MSI-L samples were included in the MSS group. When patients had multiple sequences of the tumor, we selected samples where the analyte was DNA over those derived from whole genome amplification. If there were still multiple samples, we selected the one that was sequenced later.

**Table 1:** Summary of samples analyzed in this study, including MSI-H (microsatellite instabable–high), MSS/TMB-H (microsatellite stable with high tumor mutational burden), and MSS/TMB-L (microsatellite stable with low tumor mutational burden) classifications across different TCGA datasets.

Dataset	Overall	MSI-H (%)	MSS/TMB-H (%)	MSS/TMB-L (%)	NA (%)
TCGA-COAD	403	70 (17.37%)	8 (1.99%)	325 (80.65%)	0
TCGA-READ	137	6 (4.38%)	4 (2.92%)	127 (92.7%)	0
TCGA-UCEC	509	159 (31.24%)	31 (6.09%)	309 (60.71%)	10 (1.97%)
TCGA-STAD	401	79 (19.7%)	1 (0.25%)	321 (80.05%)	0

For the TCGA-COAD and TCGA-READ datasets, we removed samples with a Gapfiller 7m target capture kit (22 samples) due to low quality.

For validation, we used the TCGA-UCEC and TCGA-STAD projects. The TCGA-UCEC had 10 samples with unidentified MSI status.

### 3.2 Tumor mutational burden

The TMB values were computed using the tmb function in maftools Mayakonda et al. (25). Sequence capture kits vary across samples in the TCGA datasets, so capture size was calculated individually for each sample based on kit information. By default, the tmb function discards silent mutations, but it has been shown that synonymous mutations play an important role in cancer Sharma et al. (26). Therefore, we included all types of mutations in our analysis.

Subsequently, the samples were categorized into three groups: MSI-H, MSS/TMB-L (MSS samples with low TMB), and MSS/TMB-H (MSS samples with high TMB) (**Figure 1**). While previous studies have used median TMB values for classification, this often results in a lower threshold that may not sufficiently capture the subset of samples with significantly higher mutational burdens. To better differentiate these samples, we adopted a TMB *>* 50 mut/Mb threshold for the TMB-H group.

### 3.3 Data quality assurance

Due to potential batch effects reported in previous studies, particularly in relation to STR mutations and capture kit variability Xia et al. (27), we evaluated the effect of sequencing plate, capture kit, age, cancer stage and clinical parameters on TMB values (**Supplementary Figures S1**–**S7**). We did not find a significant difference in this parameters between the MSI-H, MSS/TMB-H, and MSS/TMB-L groups.

### 3.4 Statistical analysis

For all statistical tests and analyses in this study, including those presented in the plots, we employed the ANOVA test followed by the Tukey’s honestly significant difference (HSD) *post-hoc* test. This approach was used to determine if there were significant differences in different parameters among the MSI-H, MSS/TMB-H, and MSS/TMB-L groups.

We used the following thresholds for p-values to indicate levels of significance: ****: p<0.0001, ***: p<0.001, **: p<0.01, *: p<0.05 Non-significant results (*p* ≥0.05) are not shown on the plots.

### 3.5 Mutational signature

To characterize mutational signatures, we used the R package sigminer Tao et al. (12). Initially, we counted mutations by specific nucleotide changes (e.g., A > C, C > T) across samples. Due to the complexity and volume of these data, we extracted five signatures per sample using the sig extract function (**Figure 2A**).

We then compared these estimated mutational profiles with the COSMIC Single Base Substitution (SBS) signatures Sondka et al. (28). For each sample and for each SBS signature, we computed the exposure values. Exposure quantifies how much a specific mutational pattern (signature) is present in the mutations observed within a sample. For each sample, an SBS signature with the highest exposure was identified; their frequencies were plotted for each group MSI-H, MSS/TMB-H, MSS/TMB-L (**Figure 3**).

### 3.6 Immune cell estimation

To assess immune cell infiltration, we used information from image data provided by Saltz et al. (19), who analyzed tissue slides for tumor-infiltrating lymphocytes (TILs). In addition to TILs, we included estimates for leukocytes, dendritic cells, and neutrophils.

Another method of estimating immune cell infiltration is based on gene expression data, as implemented in the ESTIMATE algorithm Yoshihara et al. (20). The advantage of this algorithm is its ability to analyze bulk RNA-Seq data. We used the implementation in the tidyestimate R library Aragaki et al. (29). It should be noted that ESTIMATE scores can only be interpreted relatively. Gene expression data from RNA-seq was obtained from the GDC Data Portal Heath et al. (30). We used FPKM (Fragments Per Kilobase per Million mapped fragments) values as input for the ESTIMATE method.

### 3.7 Identification of neoantigens

Human Leukocyte Antigen (HLA) class I information for the TCGA-COAD and TCGA-READ datasets was obtained from an existing study Roudko et al. (31). Common neoantigen, which are neoantigens that reoccur across different samples of colorectal cancer, was taken from study Chen et al. (23).

To identify potential neoantigens for each sample individually, we used the pVAC-seq pipeline (v. 4.0.7) Hundal et al. (24). Input VCF (Variant Call Format) files, which contain detailed information about mutations, were prepared based on the TCGA MAF (Mutation Annotation Format) files using the vcf2maf tool (v. 1.6.21) Kandoth (32). Each VCF file was then annotated with the Variant Effect Predictor (VEP) tool (v. 111.0-0) McLaren et al. (33), with options, required by the pVAC-seq tool.

To identify potential neoantigens, we analyzed peptides of length 8, 9, and 10 amino acids in the colorectal cancer datasets (TCGA-COAD and TCGA-READ). For the UCEC and STAD datasets, we restricted the analysis to 9-mers, as most HLA class I molecules display a strong binding preference for peptides of this length Andreatta and Nielsen (34). To evaluate whether this restriction is representative, we compared the number of 9-mer neoantigens per sample with the combined counts of 8-, 9- and 10-mers per sample using Spearman correlation. The resulting high correlation (R = 0.986) confirmed that the 9-mer restricted analyzes capture the relative dynamics between the groups (**Supplementary Figure S18**).

Each peptide was analyzed with a list of tools: MHCflurry, NetMHC, NetMHCcons, NetMHCpan, NetMHCpanEL, PickPocket, SMM and SMMPMBEC O’Donnell et al. (35)Reynisson et al. (36)Karosiene et al. (37). These tools predict the binding affinity of peptides to Major Histocompatibility Complex (MHC) class I molecules. We did not analyze binding to MHC class II, as it was beyond the scope of our interest and since the available algorithms for binding prediction are less reliable.

For SNPs, we only analyzed missense mutations, as these are the main variant type supported by pVACseq for neoantigen prediction.

We used default pVACseq filtering options, which report candidate neoantigens with mutant peptide binding affinity (IC50) below 500 nM. When multiple tools returned predictions for the same peptide-HLA pair, we used the median IC50 value across all tools (–top-score-metric median) for filtering and ranking. Only peptides with a mutant to wild type (MT/WT) IC50 ratio greater than 1 were included. Only somatic variants marked as PASS in the VCF were considered.

For both methods for neoantigens identification we included information on gene expression, filtering out peptides without detectable expression, defined at< 1.6 transcripts-per-million (TPM). This threshold was chosen to be the same as the one used in the study on immunity in TCGA Thorsson et al. (38).

## 4 Discussion

In our study, we focus on characterizing MSS/TMB-H tumors to understand whether they may respond well to immunotherapy.

While previous studies have linked TMB, POLE mutations, and response to immunotherapy, our work focuses specifically on MSS samples with very high TMB. We show that using a higher TMB threshold better separates hypermutated tumors driven by POLE mutations. We also integrate mutation types, neoantigen quality, immune signatures, and mutational signatures to provide a more detailed view of this group. Finally, we extend the analysis to other cancer types, showing that similar patterns exist beyond colorectal cancer.

We were able to show that MSS/TMB-H tumors have a high neoantigen load. Importantly, this is not limited to overall quantity, as we also observed a high proportion of neoantigens with strong predicted binding affinity and clear mutant-to-wild-type amino acid changes. This indicates that MSS/TMB-H tumors have not only high neoantigen load, but that many of these are of high quality and potentially immunologically relevant. We also found that mutations in POLE, BIRC6, DST, and CNOT1 contributed most strongly to the neoantigens in this subgroup.

We set the threshold for TMB-H as TMB *>* 50 mut/MB. Our reasoning was to use a value higher than the median TMB, as previous studies have shown that tumors with TMB< 12 mut/MB are associated with a lack of response to immunotherapy, while TMB levels higher than this threshold were not necessarily correlated with better outcomes Bortolomeazzi et al. (39).

We found that high amount of mutations in MSS/TMB-H tumors can be explained by POLE mutations. While Fabrizio et al. (8) found POLE mutations in only about 20% of MSS/TMB-H cases, that study assigned TMB-H status to samples with TMB *>* 11.7 mut/MB. In contrast, in our study TMB values in MSS samples with POLE mutations were significantly higher, starting from *>* 73.4 mut/MB. This, together with our results, leads to a conclusion that a higher threshold is appropriate to classify samples as TMB-H.

For all cancer types considered, the datasets were split in a similar way, according to the level of TMB and the status of MSI. The STAD dataset contained only one MSS/TMB-H sample, whereas the UCEC dataset had a significantly higher number of MSS/TMB-H samples compared to the COAD and READ datasets. In UCEC dataset, we observed that some MSS/TMB-H samples arise from different mutational processes beyond just POLE mutations and some samples with POLE mutations had low TMB.

Despite some differences, the three groups we studied: MSI-H, MSS/TMB-H, and MSS/TMB-L showed similar characteristics across different datasets.

Our study has certain limitations, which also point to directions for future research. One point of consideration is the spatial distribution of immune cells, particularly lymphocytes, which play a large role in the tumor’s response to immunotherapy. It would be beneficial to study this spatial distribution using spatial transcriptomics or single-cell approaches, because in our analysis were unable to confirm high TIL% in MSI-H tumors.

Our predictions of neoantigens are computational and it would be beneficial to have functional validation (e.g., immune recognition assays) of high neoantigen presence in MSS/TMB-H group.

Another important consideration is that due to the small number of MSS/TMB-H samples, survival analyses did not show statistical difference between groups. However, we did not expect to see a difference, as patients with MSS/TMB-H tumors did not receive immunotherapy.

Furthermore, recent research has highlighted the role of short tandem repeats (STRs) in the immunotherapy response of MSI tumors Babayan et al. (40). This suggests that STRs could potentially serve as important biomarkers in MSS tumors as well, and investigating this could improve understanding of immunotherapy response in MSS/TMB-H cases.

Our findings shows that MSS/TMB-H tumors have unique features, that separates them both from MSI-H and MSS/TMB-L tumors. That supports their potential clinical relevance, though validation in larger cohorts and clinical studies will be necessary to confirm response to immunotherapy.

## 5 Conclusions

This study provides detailed characterizations of the MSS/TMB-H subgroup across multiple tumor types. The goal was to characterize the MSS/TMB-H group and determine whether these tumors have the potential to respond to immunotherapy. To achieve this, we compared three groups of samples: MSS/TMB H, MSS/TMB-L, and MSI-H, from the TCGA-COAD and TCGA-READ datasets. We found that POLE mutations were a reason for hypermutation in MSS/TMB-H tumors.

The immune signatures calculated from gene expression data suggested that MSS/TMB-H tumors may have an immune profile similar to MSI-H tumors, although this similarity in our findings appears to be primarily driven by the leukocyte fraction. We did not observe elevated levels of lymphocytes in either of the groups.

Our analysis also showed that MSS/TMB-H tumors tend to have a high number of SNPs, while MSI-H tumors have a larger number of indel mutations. Despite the difference in mutation types, the high neoantigen load in MSS/TMB-H tumors suggests that they may have the potential to respond well to immunotherapy.

Additionally, we validated our findings in the TCGA-UCEC and TCGA-STAD datasets. The TCGA-STAD dataset contained only one sample with MSS/TMB-H, but the results were consistent with our observations. The TCGA-UCEC dataset, which included a larger number of MSS/TMB-H samples, exhibited similar patterns to those seen in the colorectal datasets, further supporting our conclusions.

These findings could be useful in identifying additional patients with MSS tumors who might benefit from immunotherapy. However, to improve the reliability of these results, it will be important to validate them further using larger clinical cohorts.

## Data Availability

The original contributions presented in the study are included in the article/**Supplementary Materia**l. Further inquiries can be directed to the corresponding authors.
