# Supplementary material for: Tumor mutational burden predicts neoantigen profiles and immunotherapy response in microsatellite stable tumors across different cancer types
Source: Front Immunol. 2026 Jan 8;16:1582464. doi: 10.3389/fimmu.2025.1582464 (PMC12824007; doi:10.3389/fimmu.2025.1582464)
Supplement: Supplementary file 1 [file DataSheet1.pdf]

1 Supplementary information

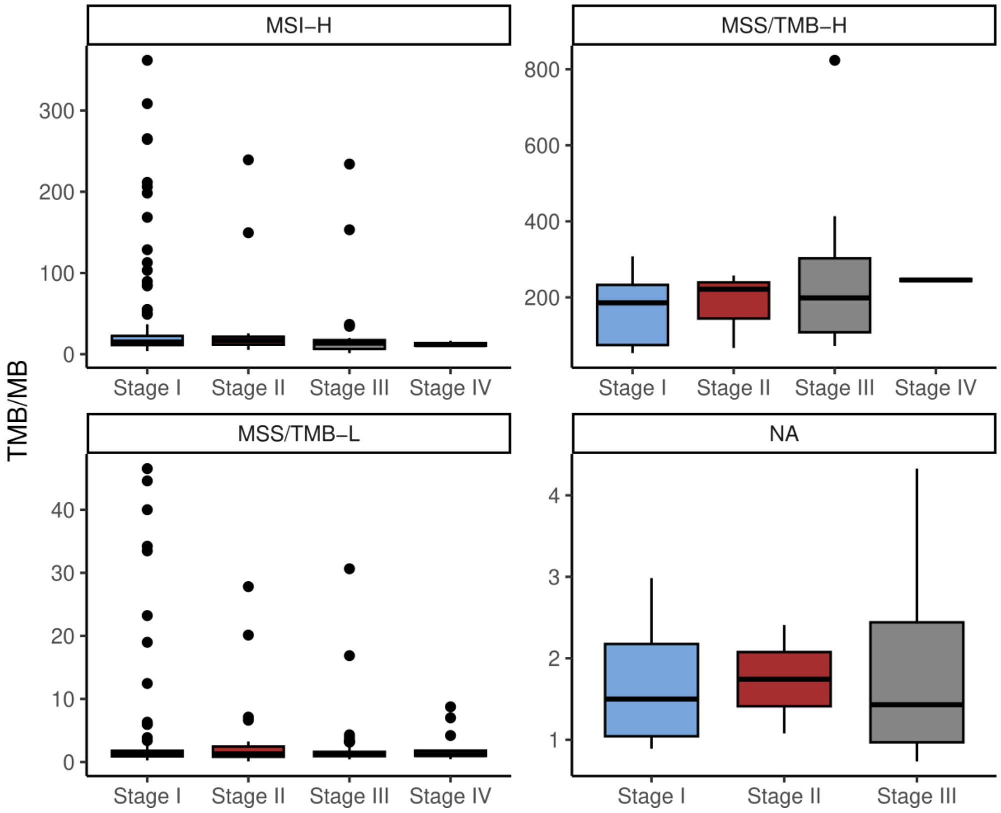

Fig. 1: Tumor mutational burden difference across various stages in TCGA-UCEC

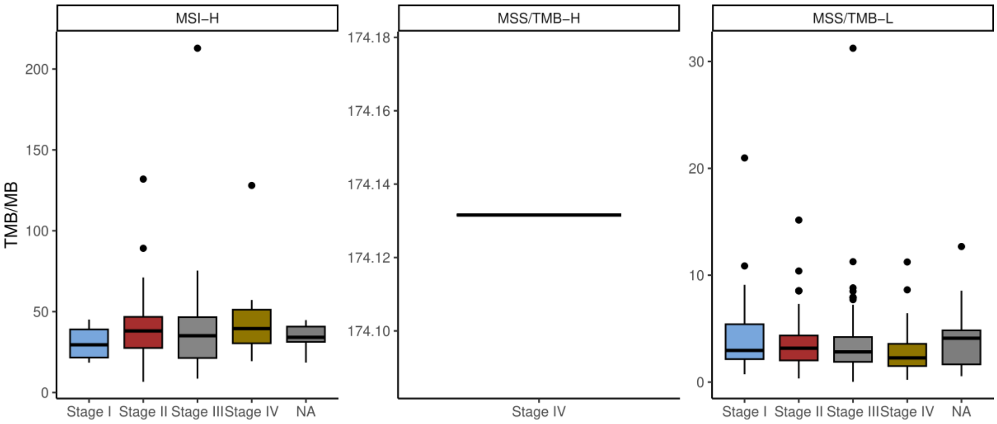

Fig. 2: Tumor mutational burden difference across various stages in TCGA-STAD

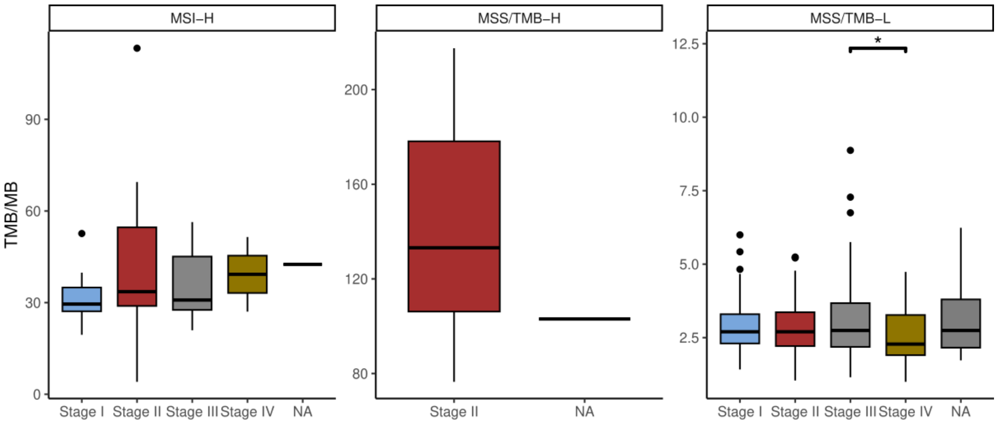

Fig. 3: Tumor mutational burden difference across various stages in TCGA-COAD

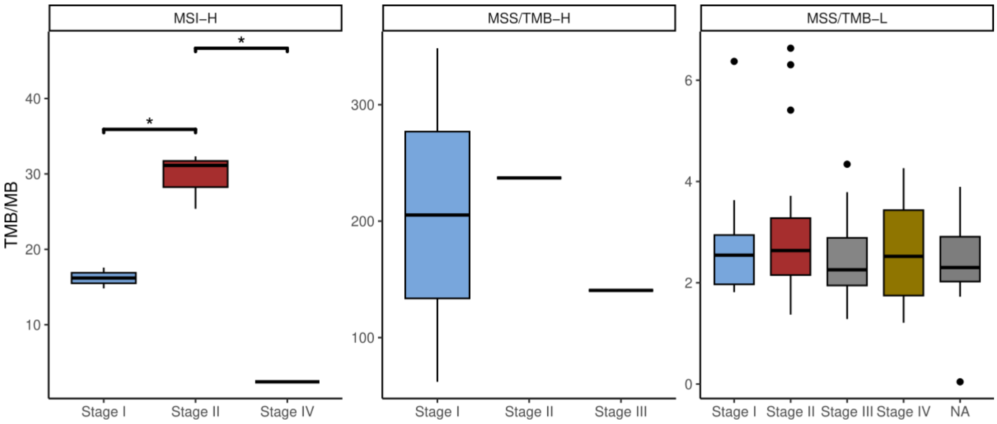

Fig. 4: Tumor mutational burden difference across various stages in TCGA-READ

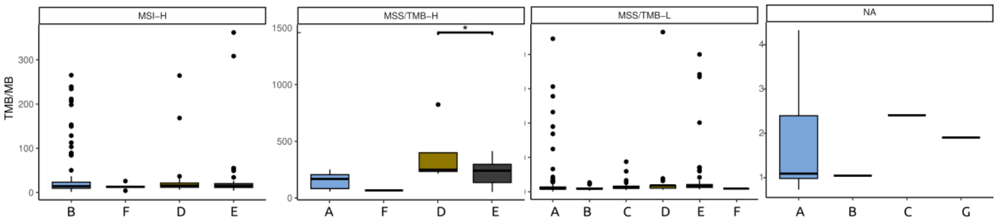

Fig. 5: Tumor mutational burden difference across various kits in TCGA-UCEC.
A: HPV_IDT_probes capture chip set — Nimblegen SeqCap EZ Human Exome Library v3.0
B: HPV IDT all pooled probes — Nimblegen SeqCap EZ Human Exome Library v3.0
C: Nimblegen SeqCap EZ Human Exome Library v2.0
D: SureSelect Human All Exon 38 Mb v2
E: hg18 nimblegen exome version 2
F: Nimblegen EZ Exome v3.0 — HPV_IDT_probes capture chip set
G: Nimblegen EZ Exome v3.0 — 120613_HG19_EC_HPV_39235 capture oligo tube — Nimblegen SeqCap EZ Human Exome Library v2.0

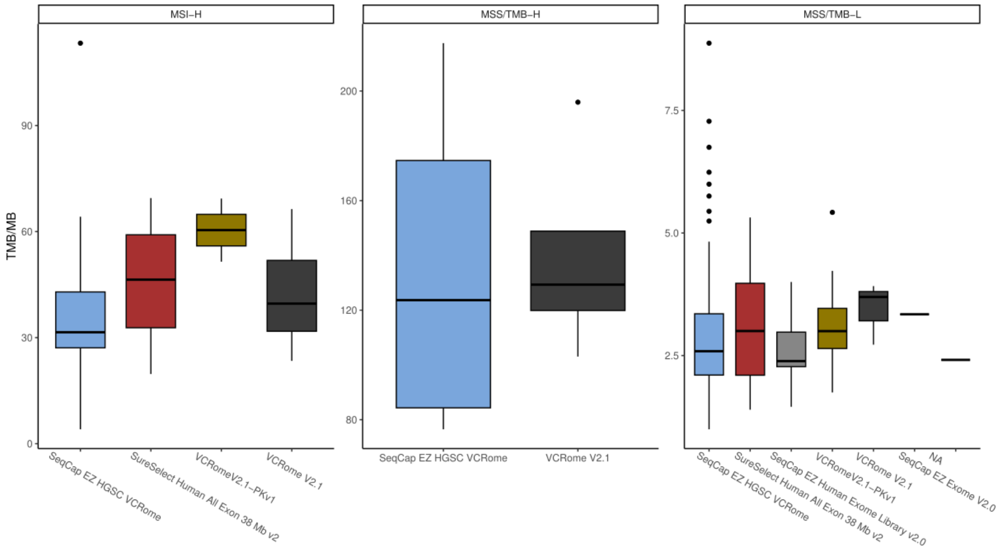

Fig. 6: Tumor mutational burden difference across various kits in TCGA-COAD

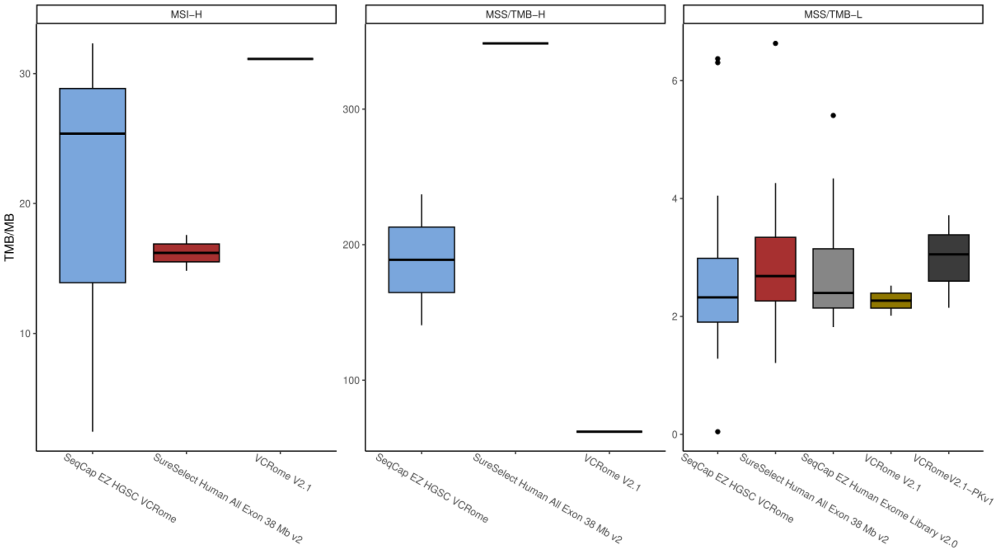

Fig. 7: Tumor mutational burden difference across various kits in TCGA-READ

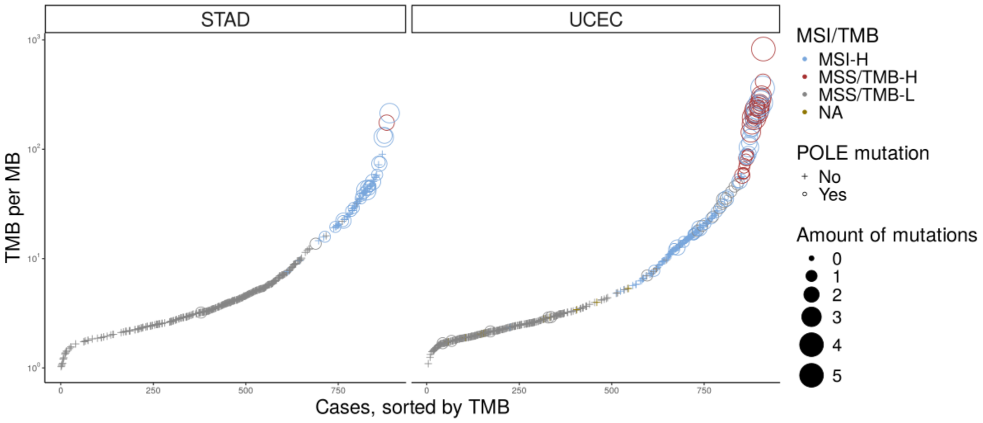

Fig. 8: POLE gene mutations in TCGA-UCEC and STAD datasets.

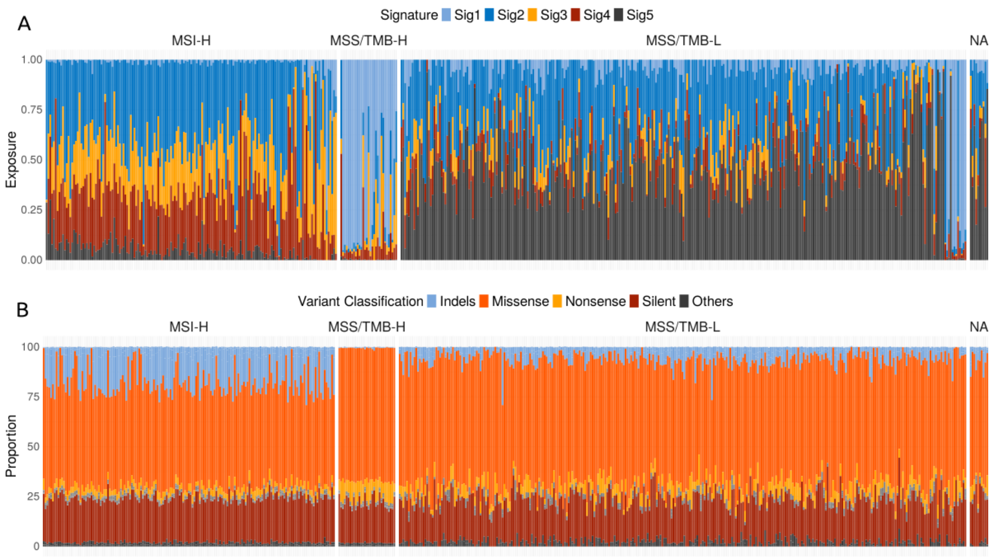

Fig. 9: TCGA-UCEC: **A** - 5 extracted signatures for MSI-H, MSS/TMB-H, MSS/TMB-L groups for each sample. **B** - Proportion of different variants in each sample.

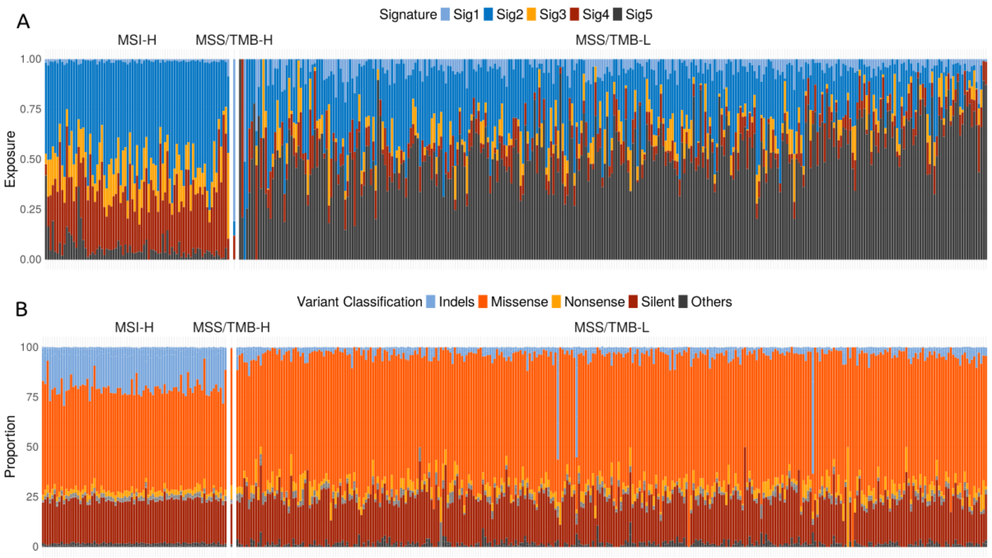

Fig. 10: TCGA-STAD: **A** - 5 extracted signatures for MSI-H, MSS/TMB-H, MSS/TMB-L groups for each sample. **B** - Proportion of different variants in each sample.

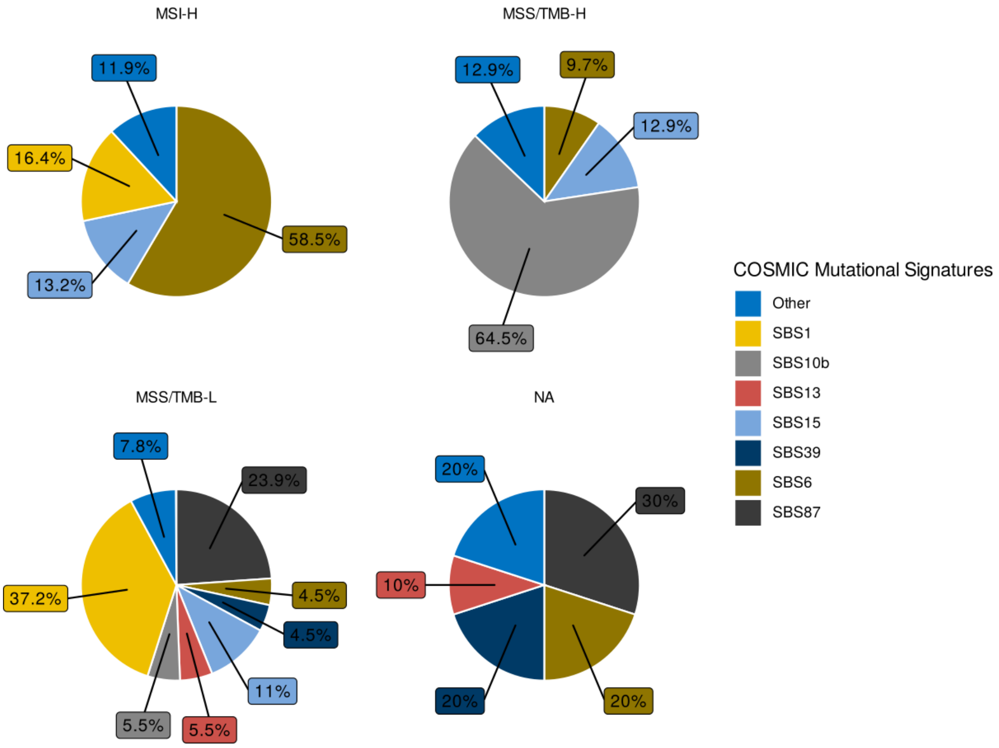

Fig. 11: Signature with highest signal in sample for each group in TCGA-UCEC.

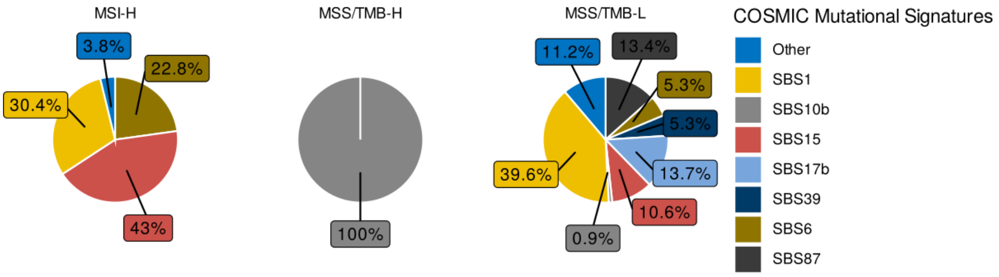

Fig. 12: Signature with highest signal in sample for each group in TCGA-STAD.

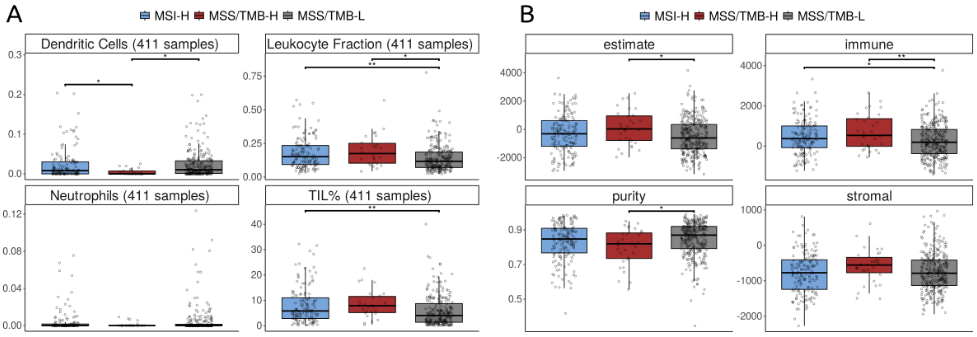

Fig. 13: TCGA-UCEC: **A** - The assessment of different cell types in the image date, **B** - A comparison of ESTIMATE Scores, where stromal is stromal signature, immune is an immune signature, estimate is score that is calculated by combining the stromal and immune scores together, purity indicates purity of the tumor.

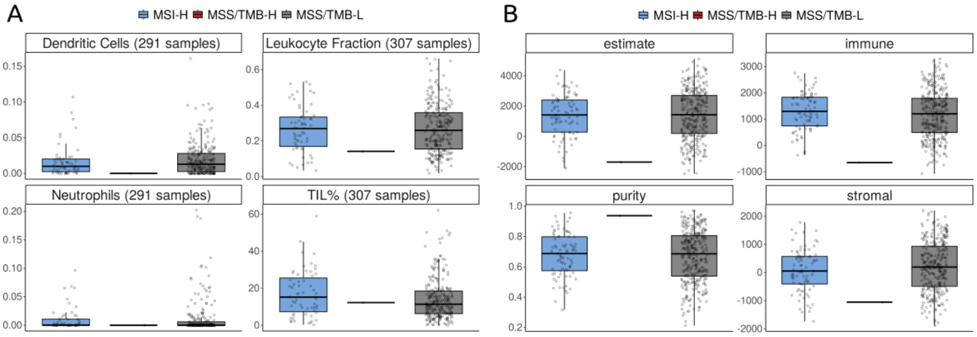

Fig. 14: TCGA-STAD: **A** - The assessment of different cell types in the image date, **B** - A comparison of ESTIMATE Scores, where stromal is stromal signature, immune is an immune signature, estimate is score that is calculated by combining the stromal and immune scores together, purity indicates purity of the tumor.

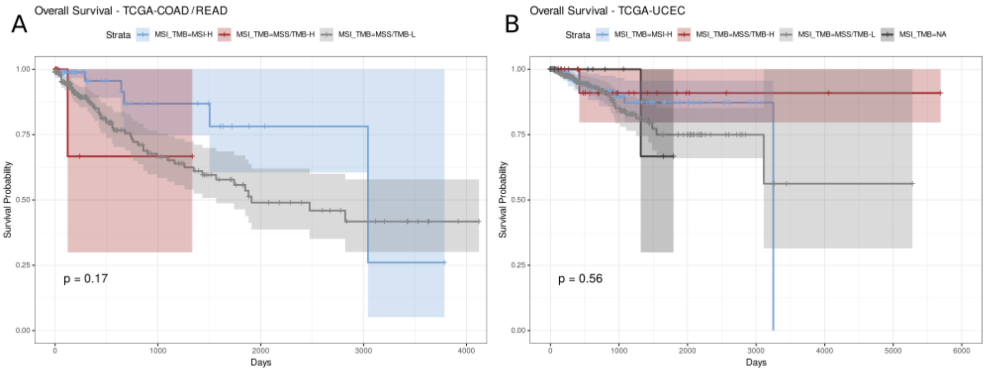

Fig. 15: Kaplan-Meier overall survival (OS) analysis across MSI-H, MSS/TMB-H, and MSS/TMB-L subgroups in TCGA datasets. **A** - TCGA-COAD and TCGA-READ combined, **B** - TCGA-UCEC.

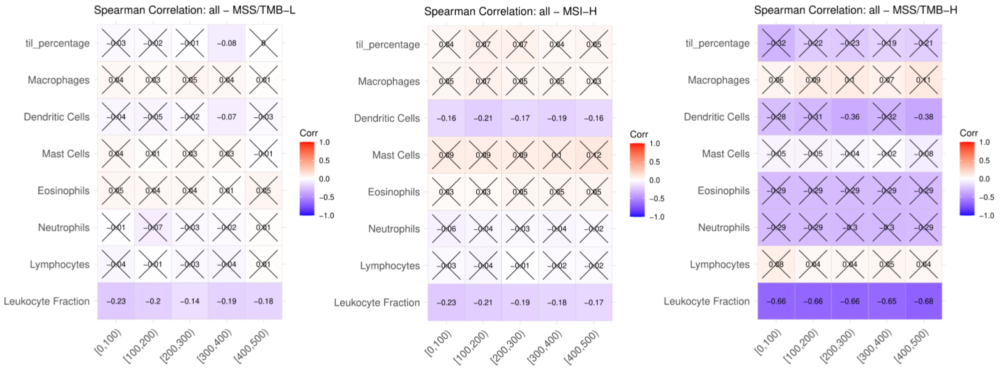

Fig. 16: Spearman correlation between neoantigen count (binned by predicted MHC-I binding affinity) and immune cell across MSS/TMB-L, MSI-H, and MSS/TMB-H groups in TCGA datasets (COAD, READ, UCEC, and STAD). Each cell displays the Spearman correlation coefficient between the neoantigen count in the corresponding IC50 bin and the specific immune cell type. Cells marked with an "X" indicate non-significant correlations (adjusted p-value > 0.05).

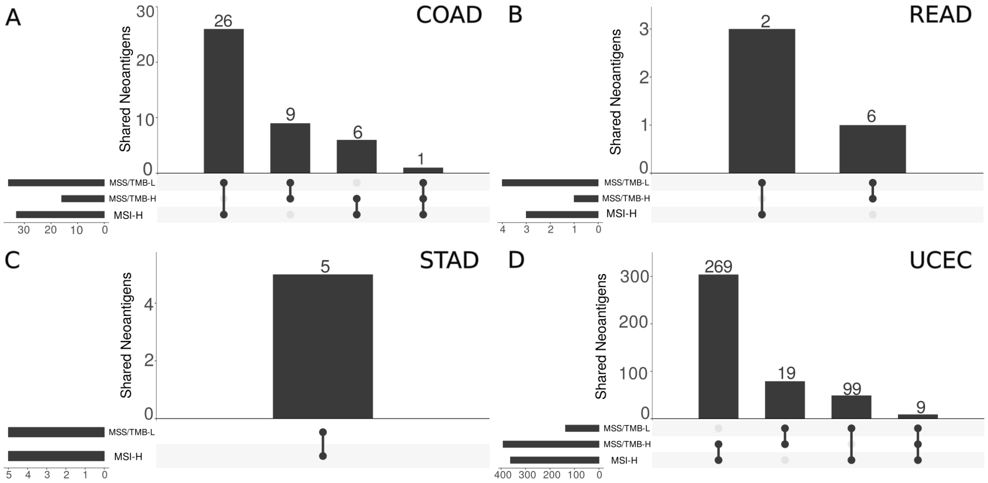

Fig. 17: Shared neoantigens across MSI-H, MSS/TMB-H, and MSS/TMB-L groups. UpSet plots of common neoantigens predicted by pVACseq in **A** - TCGA-COAD, **B** - TCGA-READ, **C** - TCGA-STAD, and **D** - TCGA-UCEC datasets. In each plot, the vertical bars (top) indicate the number of neoantigens shared between the selected MSI/TMB groups, while the horizontal bars (left) represent the total number of neoantigens that appear in other groups.

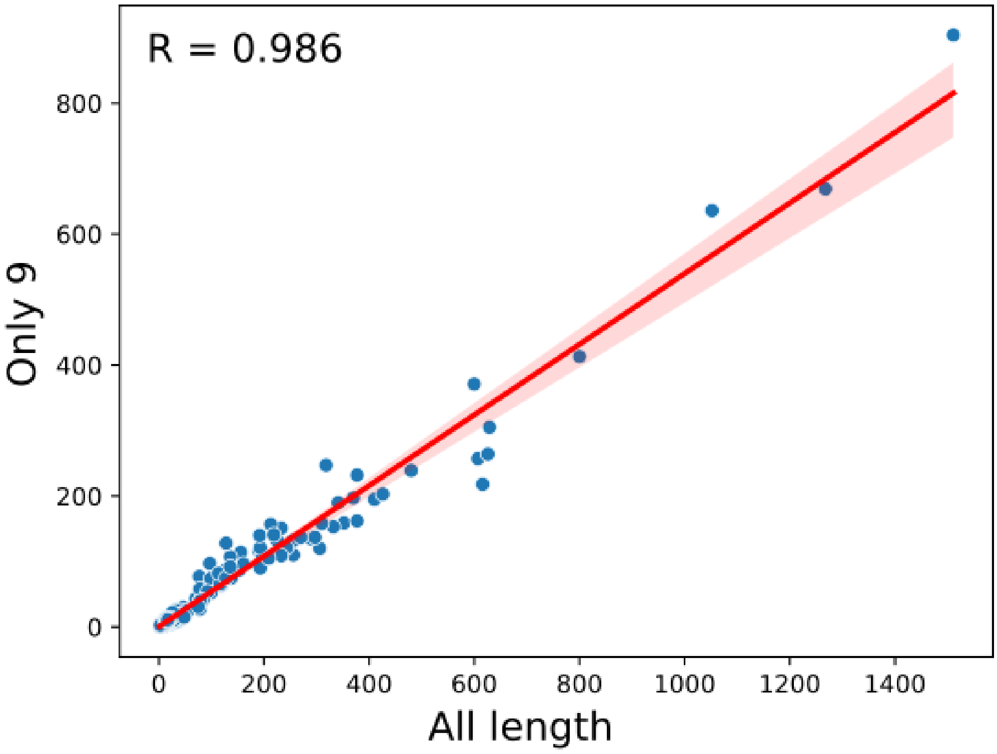

Fig. 18: The relationship between the overall number of predicted neoantigens and those restricted to peptide lengths of 9 using TCGA-COAD and TCGA-READ datasets.
